# Iodine Test for Syphilis

**Published:** 1914-08

**Authors:** 


**Iodine Test for Syphilis.** W. Landau, Presse Med., May 2, 1914, describes the following modification of his test, formerly made with a solution of iodine in mineral oil. 1-5 c.c of serum which must be free from haemoglobin, is mixed in a small tube with 1-100 c.c. oc 1% solution of iodine, in carbon tetrachlorid freshly prepared. The tube is left at room temperature, in a dark place, without shaking, for 4 hours. Syphilitic serums have a clear yellow tint, normal serums an opaque, greyish white.

